# Whole genome shotgun sequencing revealed highly polymorphic genome regions and genes in *Escherichia coli* O157:H7 isolates collected from a single feedlot

**DOI:** 10.1371/journal.pone.0202775

**Published:** 2018-08-28

**Authors:** Xin Gao, Xun Yang, Lance Noll, Xiaorong Shi, Jay Worley, Marc Allard, Eric Brown, T. G. Nagaraja, Jianghong Meng

**Affiliations:** 1 Joint Institute for Food Safety and Applied Nutrition, Center for Food Safety and Security Systems, University of Maryland, College Park, MD, United States of America; 2 Department of Diagnostic Medicine/Pathobiology, College of Veterinary Medicine, Kansas State University, Manhattan, KS, United States of America; 3 Center for Food Safety and Applied Nutrition, US Food and Drug Administration, College Park, MD, United States of America; The Pennsylvania State University, UNITED STATES

## Abstract

*Escherichia coli* serotype O157:H7 continues to pose a serious health threat to human beings. Cattle, a major reservoir of the pathogen, harbor *E*. *coli* O157:H7 in their gastrointestinal tract and shed variable concentrations of *E*. *coli* O157:H7 into the environment. Genetic characterization of cattle-shed *E*. *coli* O157 strains is of interest to the livestock industry, food business, and public health community. The present study applied whole genome shotgun sequencing (WGS) and single nucleotide variant (SNV) calling to characterize 279 cattle-shed *E*. *coli* O157:H7 strains isolated from a single feedlot located in southwestern region of the US. More than 4,000 SNVs were identified among the strains and the resultant phylogenomic tree revealed three major groups. Using the Sakai strain genome as reference, more than 2,000 SNVs were annotated and a detailed SNV map generated. Results clearly revealed highly polymorphic loci along the *E*. *coli* O157:H7 genome that aligned with the prophage regions and highly variant genes involved in processing bacterial genetic information. The WGS data were further profiled against a comprehensive virulence factor database (VFDB) for virulence gene identification. Among the total 285 virulence genes identified, only 132 were present in all the strains. There were six virulence genes unique to single isolates. Our findings suggested that the genome variations of the *E*. *coli* O157:H7 were mainly attributable to dynamics of certain phages, and the bacterial strains have variable virulence gene profiles, even though they came from a single cattle population, which may explain the differences in pathogenicity, host prevalence, and transmissibility by *E*. *coli* O157:H7.

## Introduction

*Escherichia coli* serotype O157:H7 is one of the most important foodborne pathogens and poses serious threat to human health, causing an estimated 73,480 illnesses, 2,168 hospitalizations and 61 deaths within the United States alone each year [1]. USDA/ERS estimated the annual cost of the *E*. *coli* O157:H7 cases at $271.4 million, an enormous disease burden that cannot be ignored (https://www.ers.usda.gov/data-products/cost-estimates-of-foodborne-illnesses/).

*E*. *coli* O157:H7 produces Shiga toxins and expresses type III secretion systems, which lead to a wide spectrum of diseases including the severe ones such as bloody diarrhea, hemolytic uremic syndrome (HUS), and even death [2, 3]. Infectious dose of the *E*. *coli* O157:H7 is remarkably low—as few as 10–100 colony-forming units (CFUs) may induce symptoms, and as a result, a zero tolerance policy against *E*. *coli* O157:H7 is enforced in the processed ground beef [4, 5]. Various transmission routes of *E*. *coli* O157:H7 have been proposed, and the primary one is presumably via consumption of contaminated food or water. However, in many outbreaks, the transmission details simply remained unclear [1].

Since its first report in 1982 during an investigation of hemorrhagic colitis [6, 7], *E*. *coli* O157:H7 outbreaks in the US have been repeatedly linked to raw or undercooked food of bovine origin [8–11]. Food industry has carried out extensive work to track *E*. *coli* O157:H7 along the whole beef production chain, and observed that cattle actually may harbor *E*. *coli* O157:H7 in their gastrointestinal tract but without presenting any clinical manifestations [12, 13] and the cattle fecal shedding contains viable, infectious *E*. *coli* O157:H7[14, 15]. Further epidemiology studies showed that up to 30% of the cattle would be such asymptomatic *E*. *coli* O157:H7 carriers [16, 17], which makes cattle the most important *E*. *coli* O157:H7 reservoir for the food and water contaminations. Unfortunately, few intervention measures appear effective in reduction of *E*. *coli* O157:H7 in cattle population and their immediate environments [18].

Characterization of the cattle-shed *E*. *coli* O157:H7 isolates, particularly their genome variability, transmission modes, and contamination mechanisms, is always necessitated for the interests of food safety-related trace-back investigations, risk assessment practice, and regulatory framework decision. Traditionally, *E*. *coli* O157:H7 is characterized utilizing a range of phenotypic- and/or genotypic-based typing techniques [19–23], which have significantly facilitated *E*. *coli* O157:H7 detection, clonal characterization, serotype determination, lineages differentiation, and virulence assessment [24–27]; however, the processes are generally time-consuming, expensive, and less sensitive. The advances in next-generations sequencing in the past decade have made it possible to perform whole genome sequencing (WGS) of any organism including *E*. *coli* O157:H7 rapidly and at affordable costs [28, 29]. More importantly, mining the WGS data allows simultaneous examination of multiple groups of featured gene markers, and consequently has a discriminatory power superior to any of the existing traditional methods [30, 31]. There is an ever-increasing body of evidence demonstrating that WGS has become a dominant tool in foodborne pathogen and infection investigations [32].

WGS studies on the *E*. *coli* O157:H7 have been carried out, but mostly for prevalence investigations and usually small sample collections. In the present study, we applied WGS and single nucleotide variant (SNV) analysis to 279 cattle-shed *E*. *coli* O157:H7 strains and focused on their genetic diversity and the subsequent effects. The strains were isolated from cattle feces collected from a single feedlot in southwestern region of the US. This was a large single-location collection of *E*. *coli* O157:H7. We analyzed those strains together with the reference *E*. *coli* O157:H7 Sakai genome, and other *E*. *coli* O157:H7 assemblies available from the GenBank to explore a deep phylogenetic relationship among those strains. The identified SNVs were annotated to the reference *E*. *coli* O157:H7 Sakai genome and the highly variable genomic regions and gene-set were revealed. The WGS data were further profiled against a comprehensive virulence factor database which contained more than 26,000 records, for virulence gene identification. Our results presented valuable information and novel insights for understanding the *E*. *coli* O157:H7 genome and virulence genotype evolution.

## Methods

### Bacterial strains

*Escherichia coli* strains included in the study were isolated from feces collected from 576 cattle housed in 48 soil-surfaced pens (40 ft. ×115 ft. with 36 ft. of bunk space) in a feedlot of South Western US between July and October of 2015. The Institutional Animal Care and Use Committee at Kansas State University approved the research for sample collection and detection and isolation of strains that were used in the study (IACUC # 3674). All the fecal samples were collected with a plastic spoon from freshly-defecated pen-floor fecal pats with care taken to avoid ground contamination. The spoon with feces was placed into a Whirl-pak bag (Nasco, Ft. Atkinson, WI), then transferred in cold storage to the Kansas State University Pre-harvest Food Safety Laboratory.

Fecal samples were individually processed in the lab, with 2 g of the fecal sample mixed well with the 18 ml *E*. *coli* broth (EC; Difco, ThermoFisher, Waltham, MA) and then incubated at 40°C for 6 hrs. An aliquot of the enriched fecal suspensions was subjected to immunomagnetic separation (IMS) procedure with O157 IMS beads (Abraxis®, Warminster, PA). Twenty-five microliters of the O157 bead suspensions were spread-plated onto sorbitol MacConkey with cefixime and tellurite (CT-SMAC) medium, and the plates were incubated at 37° C for 20–24 hours. Up to six sorbitol-negative colonies from CT-SMAC were randomly picked, inoculated individually onto blood agar plates (Remel), and then incubated at 37° C for 24 hours. Isolates obtained from the CT-SMAC plate were tested for agglutination for O157 antigen by latex agglutination, and if positive, tested for indole production. Isolates positive for agglutination and indole were tested by a six-plex PCR assay that targeted *rfb*E_O157_, *fliC*_H7_, *eae*, *stx*1, *stx*2 and *ehx*A genes [33]. Confirmation of *E*. *coli* O157:H7 was based on a gene profile positive for *rfb*E_O157_, *eae*, *fliC*_*H7*_ and at least one Shiga-toxin gene (either *stx*1 or *stx*2). Confirmed isolates were stored in CryoCare™ beads (Key Scientific Products, Round Rock, TX).

### Preparation of genomic DNA for high-throughput whole genome sequencing

*E*. *coli* O15:H7 strains were re-streaked onto blood agar and then subcultured in Tryptone Soy broth. The genomic DNA was extracted from the broth culture using DNeasy Blood and Tissue Kit with the QIAcube robotic workstation (Qiagen, Germantown, MD). The multiplexed genomic libraries were constructed using Nextera XT DNA Library Preparation Kit and MiSeq Reagent Kits v2 (500 Cycles, Illumina, Inc.) following the manufacturer’s instruction. Whole genome shotgun sequencing was performed using an Illumina MiSeq platform (Illumina, San Diego, CA) to generate paired-end reads.

### Sequence analysis

Raw sequencing data were subjected to *De novo* genome assembly using SPAdes 3.6.0 [34] with the default settings, and strains with spurious assembly size removed. The raw reads data from the remaining strains were first processed using the *in-house* script wrapper to remove the reads of low quality (base score cutoff: 20), low complexity (dust score cutoff: 7) and short length (length cutoff: 150) [35, 36].

The raw-read sequencing files with a calculated sequencing depth greater than 20X from the processed data were retained for the SNV call using the kSNP3 algorithm [37] and phylogenetic tree construction using FastTree (included in the kSNP3 package). SNV annotation was performed using the SnpEff script [38]. The clade classification followed the Manning system [39] using the BLAT aligner with 100% identity [39, 40].The virulence profiling was performed using the biobakery-ShortBRED package [41] against the downloaded Virulence Factors Database (VFDB) peptide sequences (www.mgc.ac.cn/Vfs) [42], which contained more than 26,000 records from various pathogen sources. All the statistical graphs were created in R.

### Dataset accession

Individual sequence data are available at the SRA, which is hosted by the NCBI, under project number PRJNA437074. Review link to the metadata: ftp://ftp-trace.ncbi.nlm.nih.gov/sra/review/SRP134008_20180307_125217_37d5c0b6b354bc3c790d2696b42756c9. Individual sequence data are available at GenBank with accession number: SRP134008.

## Results

### SNV analysis and SNV-based Phylogenomic structure characterization

The 279 *E*. *coli* O157:H7 strains used in this study originated from the fecal samples of 525 crossbred cattle housed in a single feedlot. All the sequencing data passed our *in-house* quality criteria. To decipher the high-resolution phylogenomic structure of the strains, we applied the WGS, followed by k-mer based SNV analysis [37]. A fully closed genome of *E*. *coli* O157:H7 Sakai strain (GCA_000008865.1_ASM886v1) was downloaded from the GenBank and included in the study for comparison and SNV annotation. The analysis called a total of 4,047 SNVs (1,798 to 2,406 SNVs for individual strains, S1 Table). Interestingly, none of the SNVs were present in all the analyzed genomes. Absence of the common SNVs, across all of the analyzed strains (core SNVs) indicated that indel events might be important during the evolution of the *E*. *coli* O157:H7 genome.

**Fig 1:**
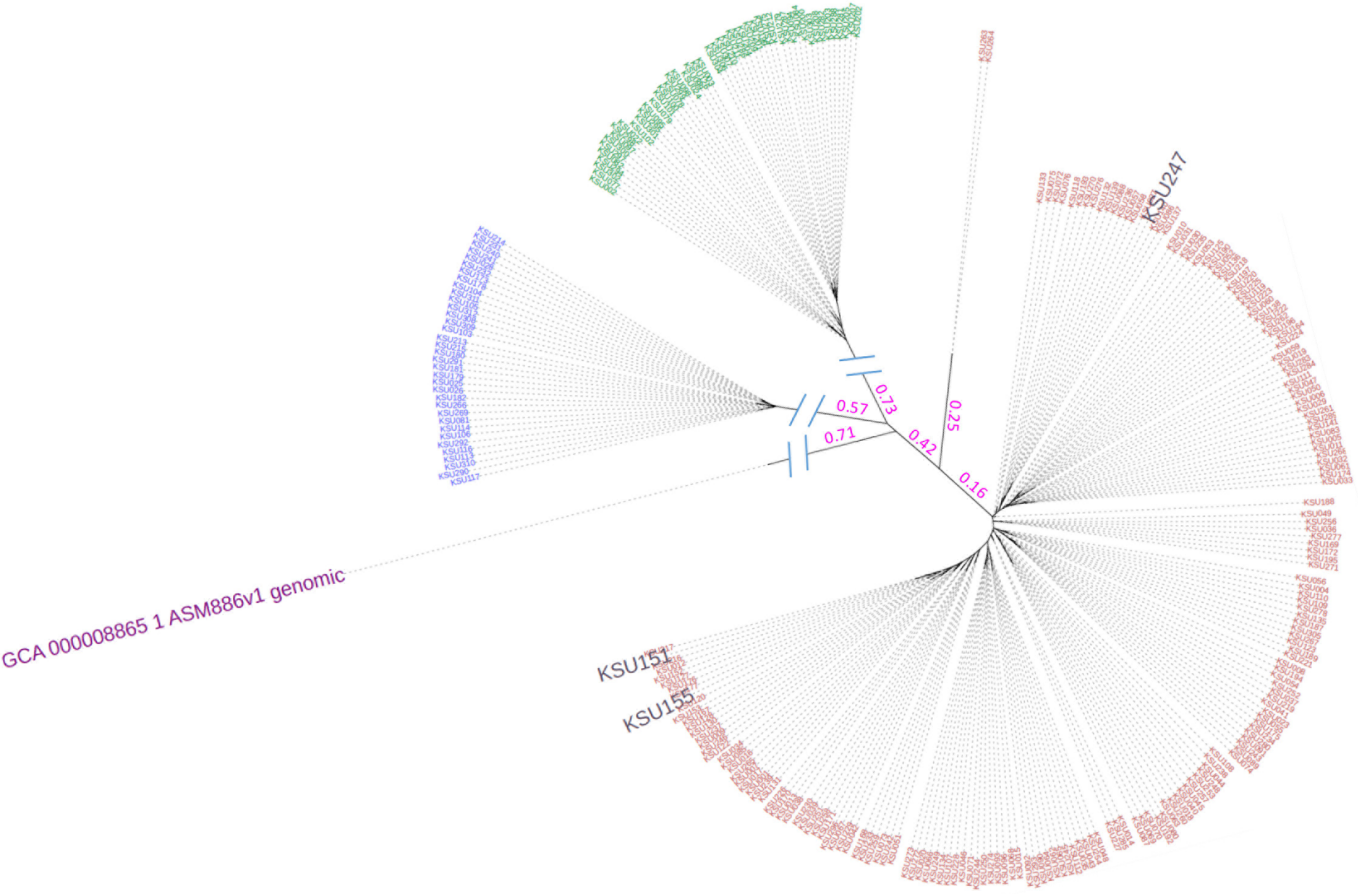
Phylogenomic relatedness of the *E*. *coli* O157 strains isolated from cattle feces from a single feedlot. ML phylogenetic tree; red, strains in clade 6; green, strains in clade 7; blue, strains in clade 8; rosy-brown, strains that could not be assigned to a clade; and purple, *E*. *coli* O157:H7 Sakai strain for reference.

With the Sakai strain genome included, we found that 2,274 SNVs (56.2%) were derived from the k-mers that were uniquely mapped to this reference, 126 SNVs (3.1%) from the k-mers mapped twice, and 1,648 SNVs (40.7%) from the k-mers not mapped at all. Such high percentage of the unmapped SNV-containing k-mers suggested that our *E*. *coli* O157:*H7* collection was quite divergent from the reference Sakai strain. The identified SNVs were used for phylogenomic tree construction (Fig 1, S1 Text). The resultant tree topology revealed 3 distinct clusters, which roughly correspond to groups 6, 7, and 8, defined by Manning Clade system (S2 Table) [39]. The clade 6 strains accounted for the largest number of the total collection (n = 191, 68.5%), followed by those belonging to clade 7 (n = 50, 17.9%) and clade 8 (n = 35, 12.5%), respectively. We also found that three strains (KSU151, KSU155, and KSU247) that could not be assigned to any clade due to the absence of the clade-specific SNVs, but clustered well with the remaining clade 6 strains. Two clade 6 strains KSU263/KSU264 appeared as outliers on the phylogeny tree, as evidenced by a separate cluster with a long branch in Fig 1.

**Fig 2:**
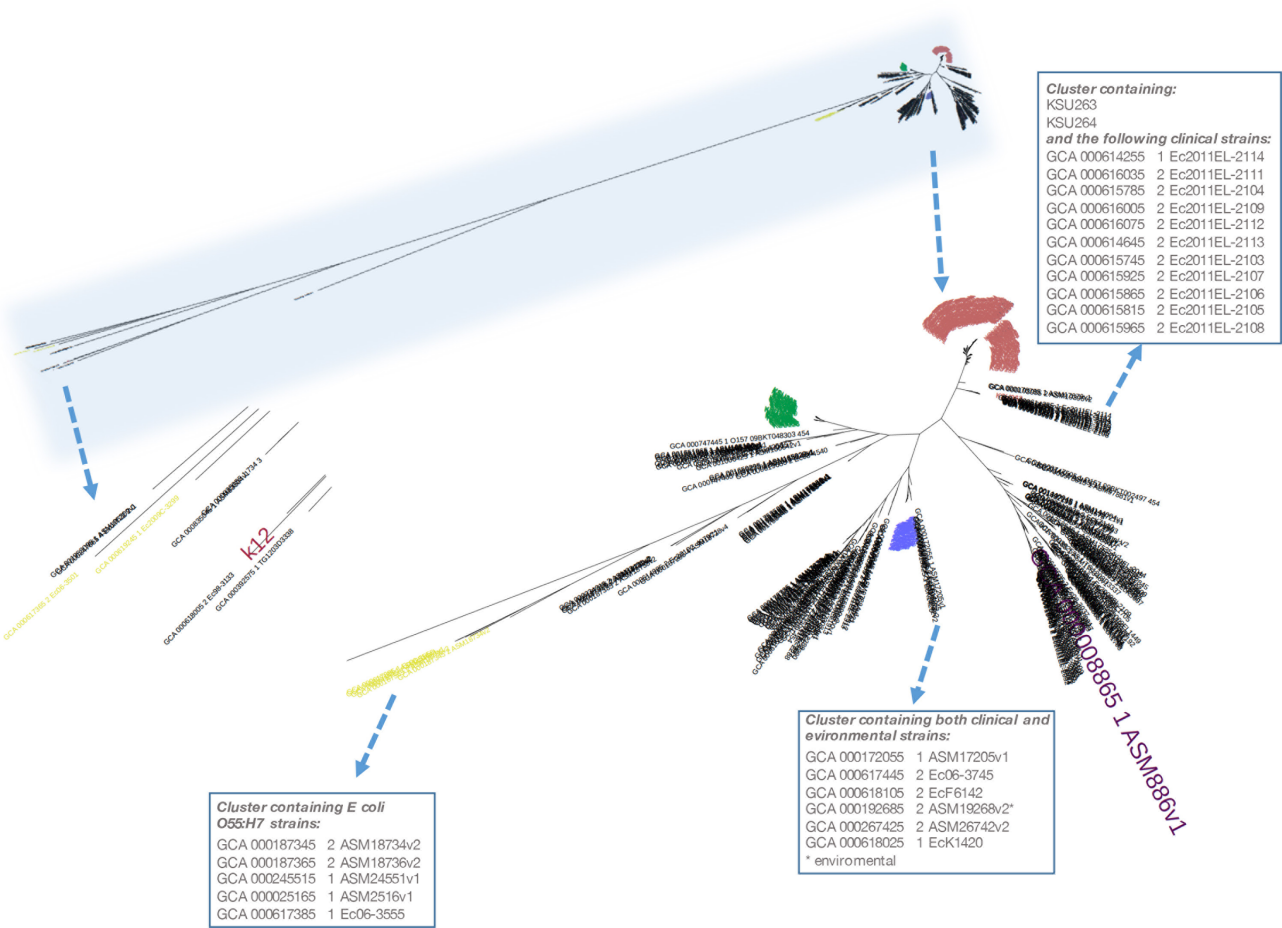
Phylogenomic relatedness among the *E*. *coli* O157 strains isolated from cattle feces from a single feedlot and the relevant genomes downloaded from the GenBank. Black, the downloaded genomes; red, strains in clade 6; green, strains in clade 7; blue, strains in clade 8; yellow, *E*. *coli* -:H7, including *E coli* O55:H7; purple, E. coli O157:H7 Sakai strain; and dark red, *E*. *coli* K12. Boxes contains the genome accession numbers corresponding to a given cluster.

To put our isolate collection into more general genetic context, an additional 175 genomes related to the *E*. *coli* O157:H7, including those either contained O157 (*E*. *coli* O157:X) or H7 (*E*. *coli* X:H7) (S3 Table), as well as one non-pathogenic *E*. *coli* K12 (GCA_000981485.1_EcoliK12AG100), were downloaded from the GenBank and subjected to another round of SNV call and tree construction (Fig 2, S2 Text). With the *E*. *coli* K12 genome used as an outgroup for the phylogeny, data demonstrated that all the *E*. *coli* O157:H7 strains tended to cluster together, consistent with the commonly accepted clonal theory of the *E*. *coli* O157:H7 population. Our outlier strains, KSU263/KSU264, stayed within the clonal *E*. *coli* O157:H7 strain cluster, but more closely related to the 11 *E*. *coli* O157:H7 strain 2011EL, all of which were clinical isolates (https://www.ncbi.nlm.nih.gov/pathogens) [43]. The data, also unsurprisingly, showed that *E*. *coli* O55:H7 strains were closely related to the *E*. *coli* O157:H7.

### SNV annotations

Since about 60% (56.2% + 3.1%) of the identified SNVs were mapped to the Sakai genome, we further performed SNV annotation to better understand how the SNVs would affect the biology of O157:H7 *E*. *coli* strains. We only focused on the SNVs that were derived from unique k-mers (n = 2,274) to avoid the interference of possible duplicated/repetitive genome regions. The SNVs occurred in both bacterial chromosome (n = 2,226) and bacterial plasmids (n = 48) (S4 Table). Among the 2,274 SNVs, 1,915 were located within the coding region including 13 that had impact on two genes due to the gene overlapping on the Sakai genome, and 359 within the intergenic region. The SNVs that were annotated into the coding regions comprised of 937 synonymous changes, 973 nonsynonymous changes, and 10 synonymous and nonsynonymous changes (dual SNVs).

The large strain collection of the present study allowed us to identify and annotate in detail a considerably higher number of SNVs compared with what has been reported before [44–47]. Using the Sakai genome, we were able to estimate that about 79% (1,796/2,274) of the SNVs had allele frequencies (AF) greater than 1%, suggesting that most of the SNVs were evolutionarily stable. SNVs were further grouped according to their AFs, and the results (S1 Fig) showed that the majority of the SNVs had AF less than 20% (n = 1,547, 68%), followed by those with AF between 60–70% (n = 243, 11%).

**Fig 3:**
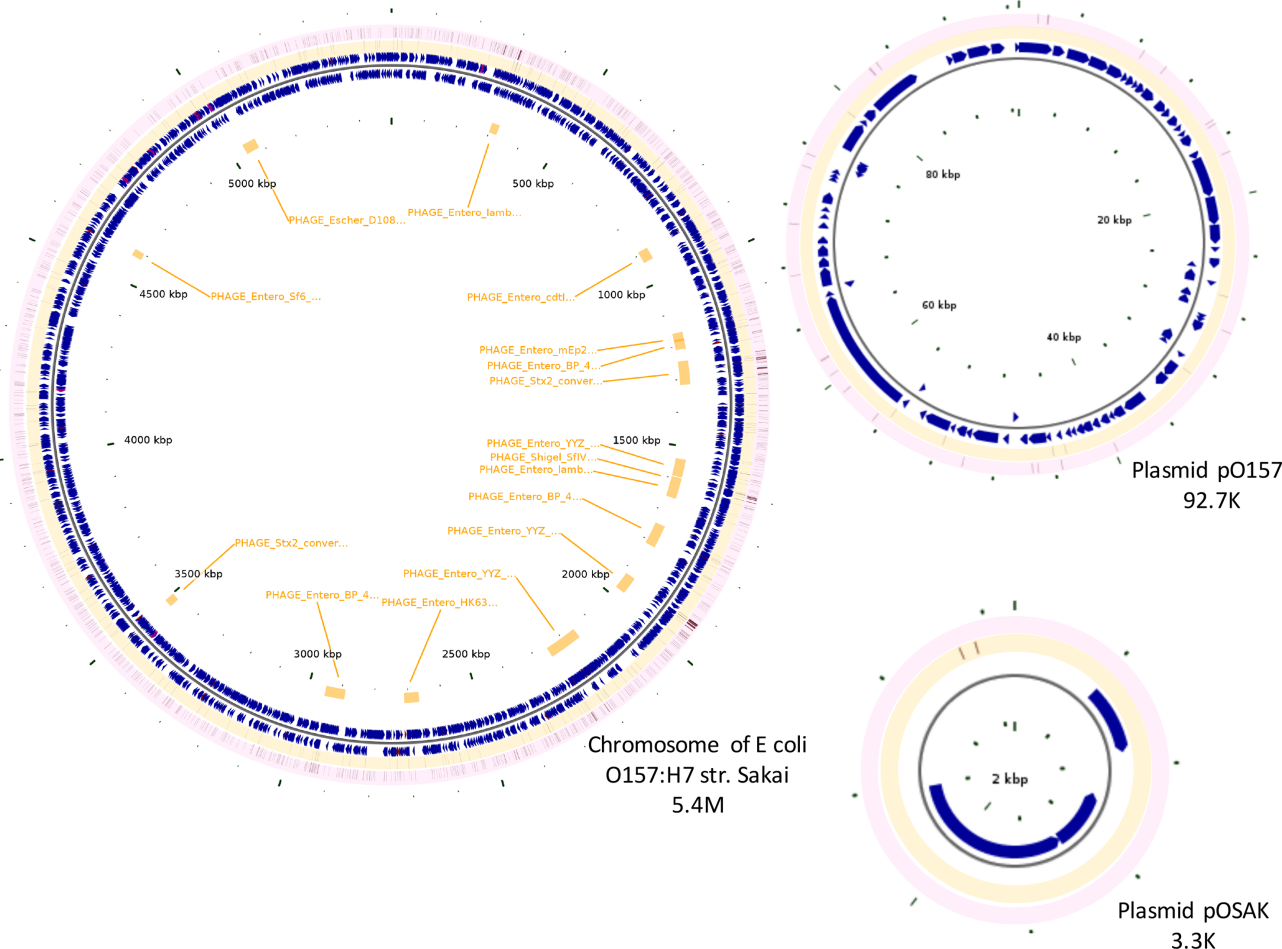
Single nucleotide variants on *E*. *coli* O157:H7 genome. Pink circular track, SNVs in genes; orange circular track, SNVs in intergenic regions; orange bars, phage regions; and blue arrows, genes.

**Fig 4:**
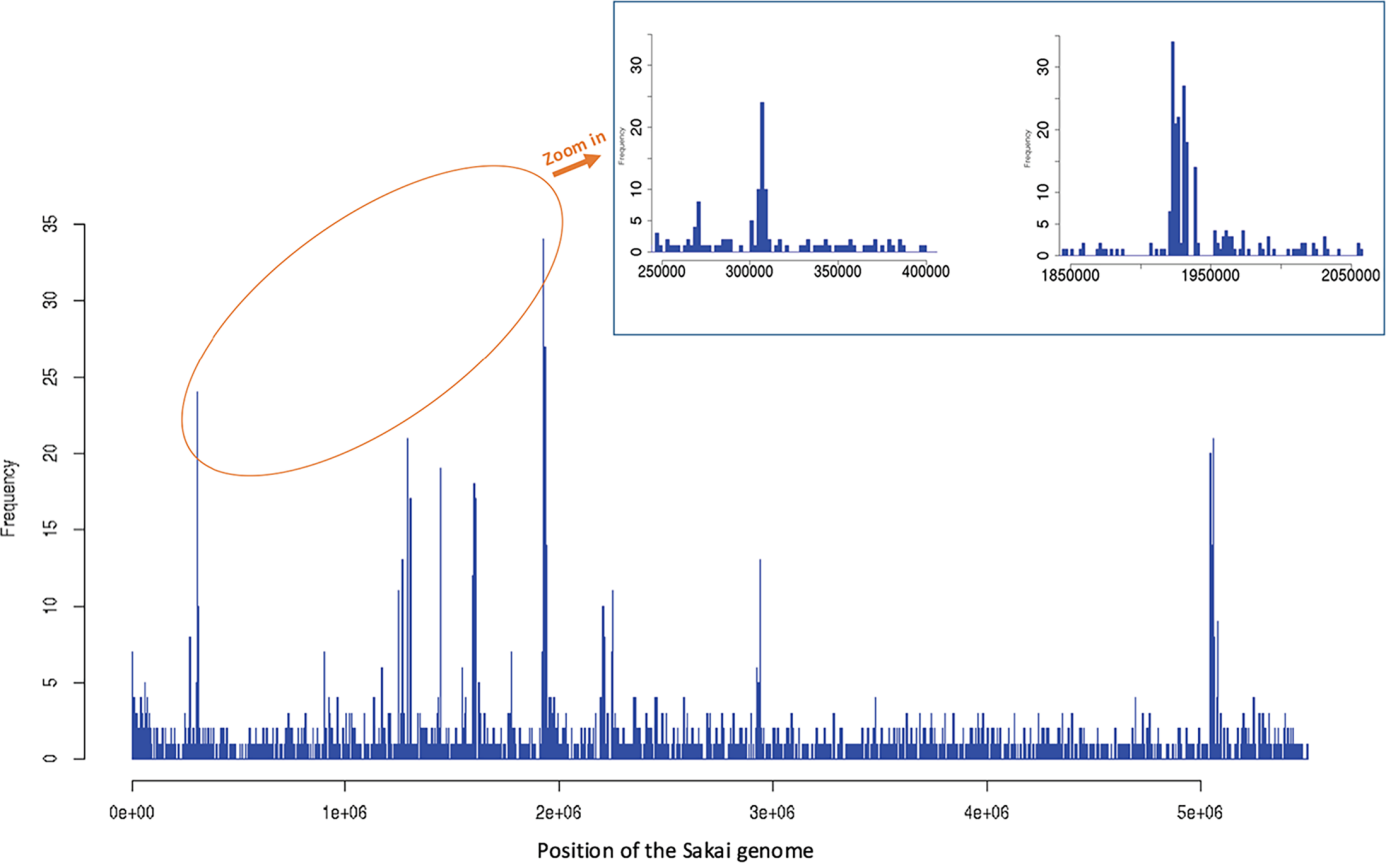
Number of single nucleotide variants (SNV) per 2, 000 bases along the *E*. *coli* Sakai genome. SNV-rich regions were zoomed in.

Fig 3 showed all the SNVs with AF greater than 1% along the Sakai genome. The track of the coding-region SNVs distinctively revealed several variant hot-spots (more than 15 SNVs within a genomic window of 2,000 bases versus an average of less than 1 SNV per 2,000-base in the Sakai genome window), as shown in Fig 4. All of the hot-spots were perfectly aligned in the prophage regions. For instance, genome regions around 1.25 M, 1.6M and 1.9 M corresponded to the prophage stx_2, prophage Shigel_SfIV, and prophage entero_YYZ loci, respectively. Instead, the distribution of the intergenic SNVs tended to be more stochastic, possibly because of overall low count (n = 359, as indicated above) of the SNVs annotated to such regions. Several intergenic regions enriched with the SNVs appeared to align within hot-spots in the coding regions.

SNVs were also annotated to the genome of non-pathogenic *E*. *coli* K12 strain AG100. When K12 was used as the genomic reference, a total of 143,463 SNVs were identified, and 92,221 of them uniquely annotated to the K12 genome. Such high number of total SNVs, when compared with that in the background of Sakai, is obviously consistent with the prominent genetic divergence between the pathogenic *E*. *coli* O157:H7 and non-pathogenic *E*. *coli* K12. It is worth noting that more than 28,000 SNVs (>35% of the annotated SNVs) presented allele frequencies greater than 0.7 as shown in (S2A Fig), strongly supporting that those genomic variants are fixed or nearly fixed within the *E*. *coli* O157:H7 genome. Comparison to the *E*. *coli* K12 strain did not reveal SNV hot-spots, indicating that divergence between the *E*. *coli* O157:H7 and *E*. *coli* K12 has no preference on specific genetic background. Even though high numbers of SNVs were found when compared with the K12 genome, short genomic stretches (genome positions: 0.3 M and 2.8 M) were SNV free and the corresponding genes may possess important house-keeping functions for bacterial survival (S2B Fig).

### Functional annotation for the genes associated with the SNVs

High-quality, deep genome sequencing in this study was key for positioning SNVs into individual *E*. *coli* O157:H7 Sakai strain genes. Out of 5,584 Sakai genes, 1,201 contained SNVs with the numbers ranging from 1 to 26, including 1,193 chromosomal and 8 plasmid genes (Fig 3, S4 Table). The SNV number for a given gene was then normalized against its length to obtain the corresponding SNV density (S5 Table). We found that 15 coding sequences possessed more than 15 SNVs per 1,000 bases (SPK, number of SNVs per 1K genome) (Table 1). Those genes were considered to be enriched for SNVs given that the genome-wise SPK was less than 1. Further analysis showed that the SNV-enriched genes were generally shorter with a mean length of only 435 bp (compared with the mean length of about 1 kb of the overall Sakai genes), and all were located on chromosomal DNA. A majority (9 out of 15, 60%) of the SNV-enriched genes coded for hypothetical proteins without specific functions assigned, and the ones that did have functional annotations (n = 6) were all involved in genetic information processing: DNA-invertase (ECs0284), transcriptional activator (ECs1588), and recombinase (ECs1933), restriction alleviation and modification enhancement protein (ECs1932), single-stranded DNA binding protein (ECs1587), and Icd-like protein (ECs1577).

**Table 1:** Genes enriched for SNVs from *E*. *coli* O157:H7.

Gene ID	Description	Length	SNV density (Counts/1000 bases)
ECs0281	hypothetical protein	593	16.86
ECs0284	DNA-invertase	554	18.05
ECs1395	hypothetical protein	872	17.20
ECs1577	Icd-like protein	197	20.30
ECs1581	hypothetical protein	299	20.07
ECs1587	putative single stranded DNA-binding protein	410	21.95
ECs1588	putative transcriptional activator	272	18.38
ECs1599	hypothetical protein	281	35.59
ECs1931	hypothetical protein	188	31.91
ECs1932	restriction alleviation and modification enhancement protein	149	20.13
ECs1933	recombinase recT protein	809	16.07
ECs1936	hypothetical protein	233	21.46
ECs1944	hypothetical protein	788	15.23
ECs1956	hypothetical protein	599	15.03
ECs1957	hypothetical protein	290	17.24

The 4,383 genes without SNVs were also examined. Interestingly, we found that 2,002 (46%) of those genes were absent in the non-pathogenic *E*. *coli* K12 strain; in contrast, the corresponding proportion was lower among the SNV-containing genes (490 out of 1,201, 41%, p value = 0.001 by fisher exact test). These results suggested that the SNV-free genes of *E*. *coli* O157:H7 *E*. *coli* genome tended to be gained from sources other than the non-pathogenic *E*. *coli* genomes during its evolution.

### Virulence gene profiling and virulent factors associated with the SNVs

Virulence factors (VFs) are essential in defining the pathogenicity of the *E*. *coli* O157:H7, which makes them the single most important gene family within the genome. Therefore, we profiled the shotgun sequencing data from our *E*. *coli* O157 strains against the 26K VFDB sequence records, using the ShortBRED pipeline. A total of 285 virulence genes were identified from our strain collection (S6 Table), and only 132 (46.3%, 132/285) of them were present in all of the profiled strains, indicating that genomes of the *E*. *coli* O157:H7 strains have dynamic and independently gained virulence genes. The common 132 virulence genes included *stx*2B and others that were characteristic to the *E*. *coli* O157:H7, such as the type III secretion system components (the Esp proteins), and the various flagellar proteins. One strain (KSU127) also contained the *stx*1B gene. Among all the identified virulence genes, 6 were only present in one of the profiled isolates (as listed in Table 2), including the *stx*1B (found in KSU127 isolate).

**Table 2:** Virulence factors that were identified in single KSU isolate.

VFDB ID	Description	KSU isolate ID
VFG000836(gb|NP_288672)	(stx1B) shiga-like toxin 1 subunit B encoded within prophage CP-933V [Stx (VF0206)] [Escherichia coli O157:H7 str. EDL933]	KSU127
VFG035574(gi:218693685)	(aec27/clpV) ATP-dependent Clp proteinase Aec27 ATP-binding chain, with chaperone activity [ACE T6SS (CVF736)] [Escherichia coli 55989]	KSU263
VFG035974(gi:15800037)	(ehaA) beta-barrel outer membrane protein [EhaA (SS109)] [Escherichia coli O157:H7 str. EDL933]	KSU055
VFG043048(gi:16765293)	(ppdD) major pilin subunit [Hemorrhagic Coli pili (HCP) (AI102)] [Escherichia coli O157:H7 str. EDL933]	KSU126
VFG043263(gi:16802741)	(flk) flagella biosynthesis regulator [peritrichous flagella (AI140)] [Escherichia coli O157:H7 str. EDL933]	KSU105
VFG034822(gi:260843778)	(espR1)T3SS effector-like protein EspR-homolog[EspR1 (CVF696)]	KSU303

**Fig 5:**
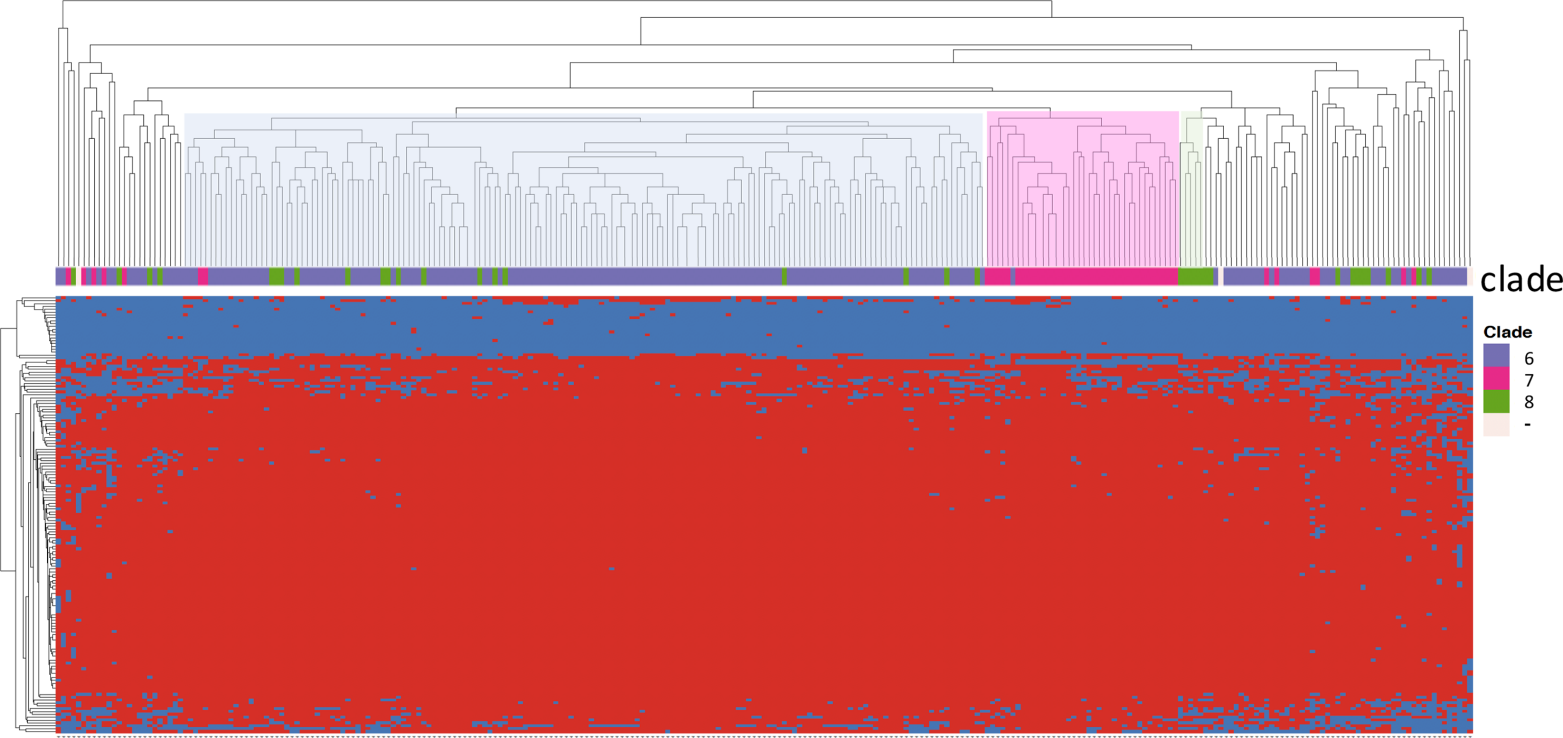
*E*. *coli* O157:H7 strains clustering based on the differences in the virulence gene profile. Clusters dominant with strains from the clades 6, 7, and 8 were highlighted in blue, pink and green, respectively.

The *E*. *coli* O157:H7 strains were clustered based on the presence of the identified virulence genes. We found that strains from clades 6 and 7 tended to cluster well with the other strains from the same clade (Fig 5). However, strains from clade 8 spread out, except for one small cluster which contained only 5 strains.

We then examined the SNV status of the identified VF genes using our SNV annotation data. We first excluded the 51 VF genes that did not have the Sakai strain counterparts. Among the remaining ones, we found that 111 possessed no SNVs and 78 strains possessed low-density SNVs (with mean SNV density of 3 per 1,000q bases), suggesting that VF genes belonged to a gene family with little genetic variances. We did observe three VF genes ECs0319 (hypothetical protein), ECs2665 (FliT), ECs1396(AidA-I), with 12, 11, and 10 SNVs per 1,000 bases, respectively. The function of ECs0319 is still unknown, the ECs2665 (FliT) acts as transcriptional regulator and molecular chaperon to stabilize the flagella, and ECs1396 (AidA-I) facilitates adherence of the *E*. *coli* O157:H7 to the host cells. Their relatively higher variability might help the pathogen to adapt to different hosts.

## Discussion

Direct link between the cattle-derived pathogenic *E*. *coli* O157:H7 and human infections, including severe complications, have been confirmed by many microbiological and epidemiological studies [48, 49]. The beef industry and the regulatory agencies have invested tremendous efforts to prevent entry of the *E*. *coli* O157:H7 into the food supply chain. A complicated issue about the *E*. *coli* O157:H7 control in cattle rearing practice and subsequent food safety management is its transient presence in asymptomatic cattle. In-depth evaluation of the genomic variability of such an important pathogen could provide insights into its pathogenic potentials and facilitate our understanding of the disease severity and search for new effective intervention solutions.

The goal of the present study was to better understand the genetic variations among the *E*. *coli* O157:H7 strains recovered from a single feedlot and immediate effects derived from those variants. We performed massive WGS on a total of 279 *E*. *coli* O157:H7 strains. With the high-quality, deep sequencing reads and available closed reference Sakai genome, we were able to profile all the SNVs across the whole *E*. *coli* O157:H7 genome region, which allowed us not only to construct the relatedness among all of the strains, but also locate the genes and the intergenic regions containing those genetic variances. Our results suggested that individual *E*. *coli* O157:H7 genome contains around 2,000 SNVs, which translate to one SNV per 2.8k bp within the genome.

The SNV-based phylogenomic tree confirmed the genetically homogeneous structure of *E*. *coli* O157:H7, and the extraordinary resolution of the tree basically clustered our *E*. *coli* O157:H7 collection into three major groups, essentially equivalent to the three defined clades [39]. We found that 12.5% of the collection was seen in clade 8 and 68.5% in clade 6, which have been reported to possess hyper-virulent phenotypes [50]. Particularly, the clade 8 is more likely to be associated with the severe disease cases. These results were quite different from those reported recently by our group based on a survey that involved 20 cattle farms from various regions in California, which revealed that the clade 8 was the most dominant group [51], probably reflecting certain geographic effects on the *E*. *coli* O157:H7 lineage distribution, as our current strains were from a feedlot located in the South Western region of the US. Two strains (KSU263/264) in our collection had distant phylogenetic relationship with all of the remaining ones and further analysis found that they were more closely clustered with the clinical O157 isolates 2011ELs. The three strains (KSU151, KSU155, and KSU247) that formed a subclonal group with clade 6 strains did not present typical clade-6 SNVs, suggesting that Manning clade classification method has its own limitations. However, it is beyond the scope of the current study to detail genome features with potential impact on their pathobiology and genetic classification.

Our SNV results did not reveal any core SNVs, and roughly 40% of the identified SNVs were actually absent from the reference Sakai genome, strongly suggesting the evolutionary plasticity within the *E*. *coli* O157:H7 clone and the high possibility of indel events during evolution. Nearly 80% of the SNVs were shown to have AF greater than 1%, indicating that the variant spectrum was stabilized. These results also provided additional evidence for the open pangenome model of the general *E*. *coli* species [52], which was inferred by gene content analysis, and highlighted the importance of *de novo* SNV calling during the evaluation of *E*. *coli* O157:H7 genome variations.

SNVs- enriched genomic windows along the Sakai genome were identified with our massive data. They were all within prophage loci. The *E*. *coli* O157:H7 Sakai genome contained 16 prophage loci with 11 of them intact [53]. Our data showed that that some of those loci were highly variable. For instance, prophage stx2_converting_II (genome region 1245903–1316397, about 70kb long) contained a total of 115 SNVs, and two genes ECs1242 (hypothetical protein) and ECs1232 (hypothetical protein) that were located at the 3’ end of this prophage contributed to 36 SNVs. Similarly, the prophage entero_YYZ_2008 (genome region 1920485–1972367, about 52kb long) had a total of 170 SNVs, and it 5’ end gene ECs1934 (exonuclease VIII RecE) alone contributed to 26 SNVs. Prophage loci contain the integrated bacteriophage components, which have been proposed to play important roles in the emergence of the *E*. *coli* O157:H7 and their virulence [54]. The higher mutational rate of such loci suggested their frequent integration, which obviously would boost the transmission of the related virulence genes.

The detailed SNV annotation allowed us to examine individual genes for SNV density (SPK). We calculated gene-wise SPK and found that *E*. *coli* O157:H7 genes generally have less than 5 SPKs with peak at 1 SPK, suggesting a general functional stability of the basic bacterial genetics, although it won’t rule out some type SNVs may drastically change the genome coding and the related biology. The small panel of highly variable genes contained many proteins with unknown functions. However, several proteins involved in DNA/RNA processing were revealed to contain high SPKs, strongly suggesting their roles in *E*. *coli* O157:H7 genome evolution and adaptation.

Clinical manifestations of the *E*. *coli* O157:H7 infections result from its virulence factors. Current VFDB collected about 26,000 VFs from various organisms. To get a comprehensive understanding about the virulence of the *E*. *coli* O157:H7 strains, we first generated a cluster-based secondary database using the VFDB peptide sequences which contained only the unique tags for any given VF gene or VF gene cluster. The secondary database allowed rapid screening of our massive WGS data against the large VFDB database records. Collectively, we found 285 VF genes among our 279 genomes. More than half of the identified VF genes (n = 153) were present in only a proportion of the strains, which might explain the wide disease spectrum of *E*. *coli* O157:H7 infections. The uneven presence of the VF genes in *E*. *coli* O157:H7 genomes also suggested that this gene family contributed significantly to the overall genomic variation among the strains. However, it is worth noting that there is a tradeoff between speed and resolution when the secondary database-based screening algorithm is applied. Some of the identified VF genes actually may represent several related VF genes.

## Conclusions

Our analyses established SNV profiles for 279 *E*. *coli* O157:H7 strains collected from a single feedlot and the majority of the SNVs have never been reported before. Although geographically limited, we observed 3 out of 9 reported *E*. *coli* O157:H7 clades in our collection. Large collections and high-quality WGS data significantly expanded our knowledge about the genomic dynamics of this important pathogen, which may be subsequently applied to other pathogenic bacteria and more diverse sample collections. The data demonstrated the power of combing the genome profiling and gene functional analysis, and should prove useful in future framework development of epidemiological and biological studies of *E*. *coli* O157:H7.

## Supporting information

S1 Fig Histogram of the single nucleotide variant allele frequencies (AF) of *E*. *coli* O157:H7 strains isolated from cattle feces from a single feedlot in when Sakai genome was used as an annotation reference. (TIF)

S2 Fig Single nucleotide variant (SNV) allele frequencies and positions of *E*. *coli* O157:17 strains isolated from cattle feces from a single feedlot when *E*. *col*i K12 strain was used as an annotation reference. (a) Histogram of the SNV allele frequencies (AF) when K12 was used as reference. (b) Number of SNVs per 2,000 bases along the Sakai strain genome. SNV-free region was zoomed in.
(TIF)

S1 Table Number of SNVs in individual *E coli* K157:H7 isolates. (XLSX)

S2 Table Clade profile for the *E*. *coli* O157:H7 collected by KSU. (XLSX)

S3 Table Genomes related to the *E*. *coli* O157:H7 downloaded from the GenBank. (XLSX)

S4 Table SNVs identified from the KSU *E*. *coli* O157:H7 isolates using Sakai strain as reference. (XLSX)

S5 Table *E coli* O157:H7 genes that contain SNVs. (XLSX)

S6 Table Virulence factors identified from the KSU *E coli* O157:H7 isolates. (XLSX)

S1 Text Tree file for the *E*. *coli* O157 strains isolated from cattle feces from a single feedlot. (TXT)

S2 Text Tree file for the *E*. *coli* O157 strains isolated from cattle feces from a single feedlot and the relevant genomes downloaded from the GenBank. (TXT)
