# Supplementary figures and images for: Whole genome shotgun sequencing revealed highly polymorphic genome regions and genes in Escherichia coli O157:H7 isolates collected from a single feedlot
Source: PLoS One. 2018 Aug 28;13(8):e0202775. doi: 10.1371/journal.pone.0202775 (PMC6112667; doi:10.1371/journal.pone.0202775)

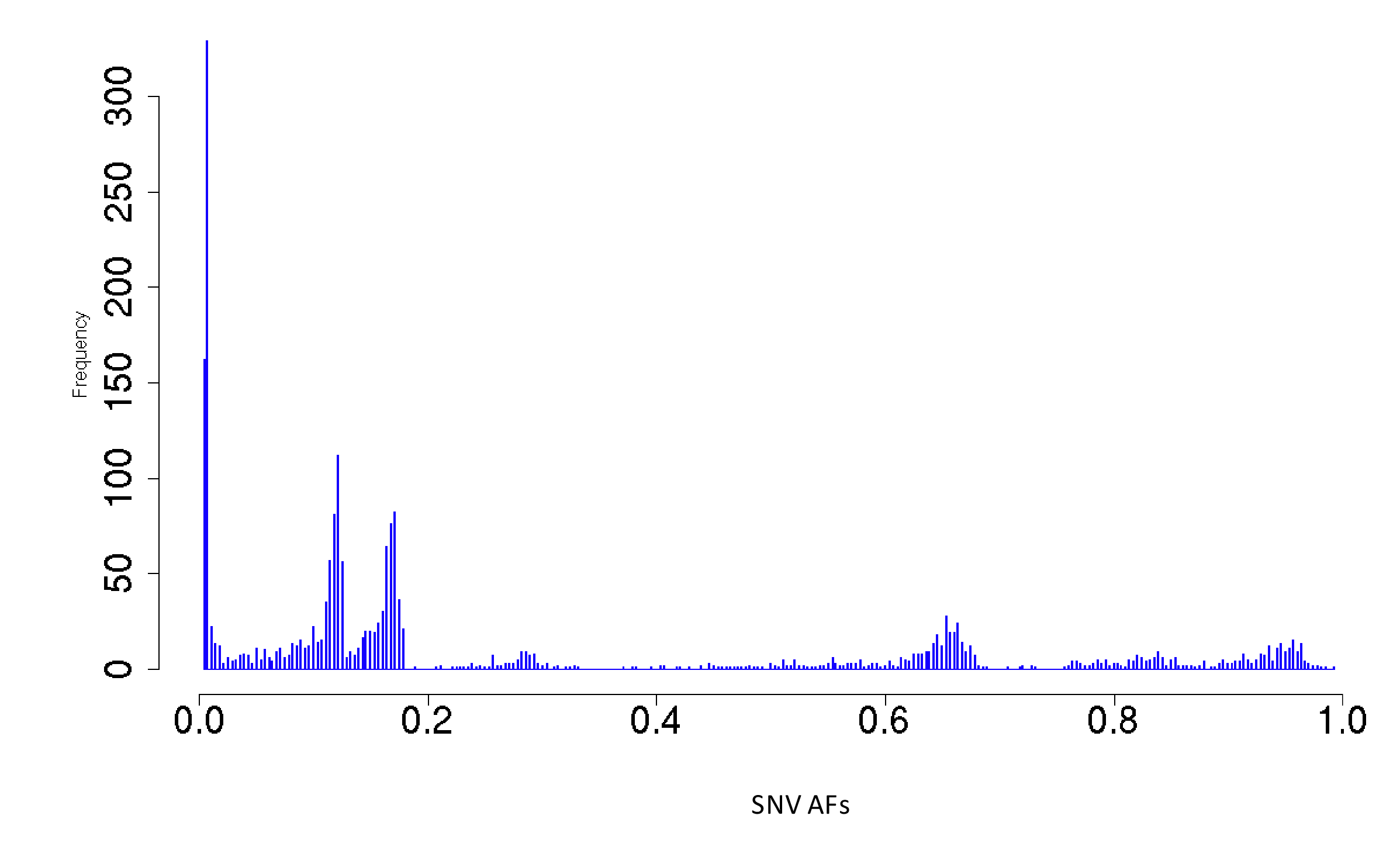

Supplement: S1 Fig — (TIF) [file pone.0202775.s001.tif]

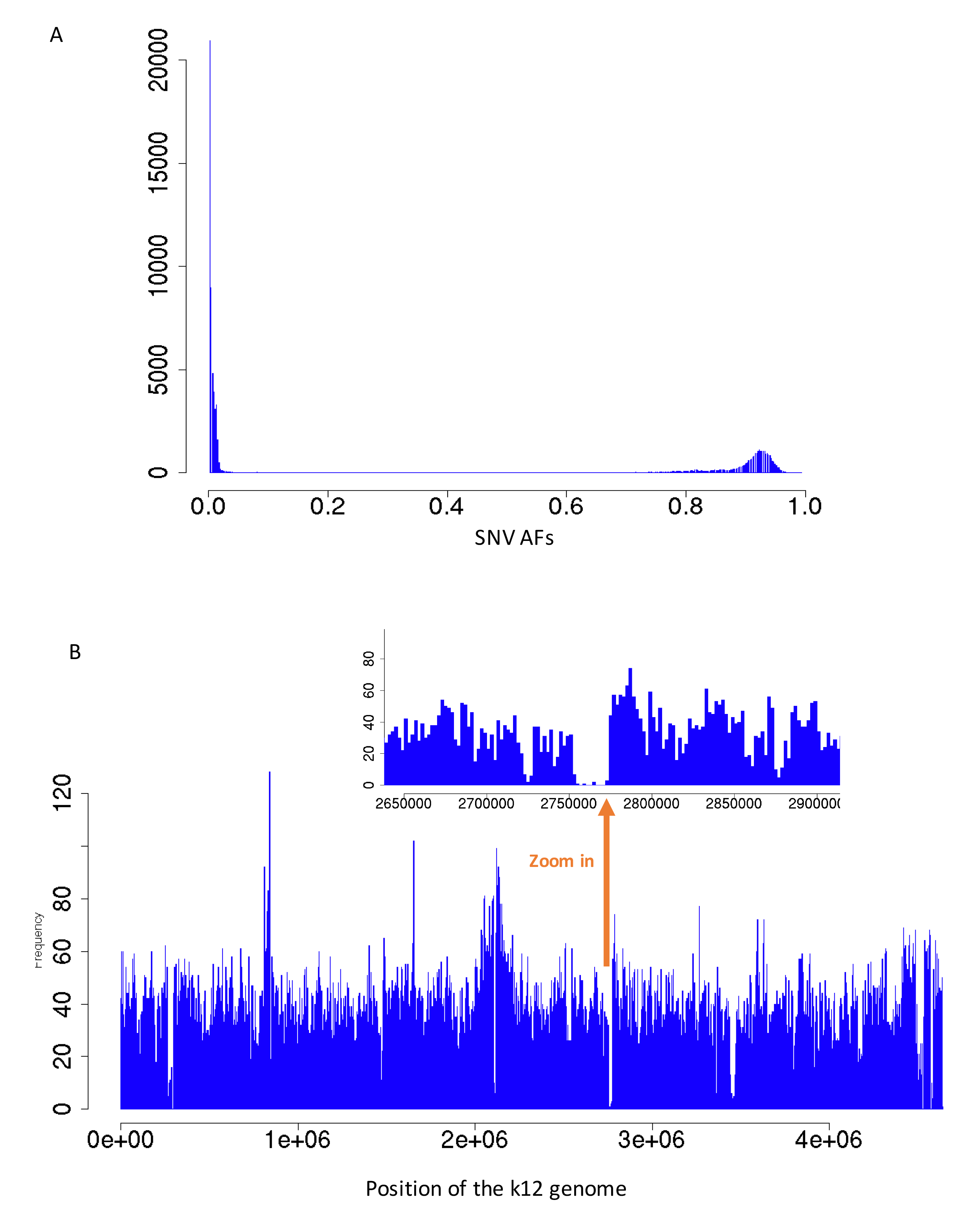

Supplement: S2 Fig — (a) Histogram of the SNV allele frequencies (AF) when K12 was used as reference. (b) Number of SNVs per 2,000 bases along the Sakai strain genome. SNV-free region was zoomed in. (TIF) [file pone.0202775.s002.tif]
